# Replicated methylation changes associated with eczema herpeticum and allergic response

**DOI:** 10.1186/s13148-019-0714-1

**Published:** 2019-08-23

**Authors:** Meher Preethi Boorgula, Margaret A. Taub, Nicholas Rafaels, Michelle Daya, Monica Campbell, Sameer Chavan, Aniket Shetty, Chris Cheadle, Sangjucta Barkataki, Jinshui Fan, Gloria David, Terri H. Beaty, Ingo Ruczinski, Jon Hanifin, Lynda C. Schneider, Richard L. Gallo, Amy S. Paller, Lisa A. Beck, Donald Y. Leung, Rasika A. Mathias, Kathleen C. Barnes

**Affiliations:** 1 University of Colorado, Denver, CO USA; 2 Department of Biostatistics, Bloomberg School of Public Health, Baltimore, MD USA; 3 Center for Patient Derived Models (CPDM), Dana-Farber Cancer Institute, Boston, MA USA; 4 Elsevier Inc, Rockville, MD USA; 5 Qiagen Sciences Inc, Frederick, MD USA; 6 Department of Medicine, Johns Hopkins University, Baltimore, MD USA; 7 Rho, Inc, Chapel Hill, NC USA; 8 Oregon Health & Science University, Portland, OR USA; 9 Boston Children’s Hospital, Boston, MA USA; 10 University of California San Diego, San Diego, CA USA; 11 Northwestern University, Chicago, IL USA; 12 University of Rochester, Rochester, NY USA; 13 National Jewish Health, Denver, CO USA; 14 University of Colorado Denver, 13001 E. 17th Place, 5th Floor East, 5330A, Aurora, CO 80045 USA

**Keywords:** Atopic dermatitis, Eczema herpeticum, Human epigenetics, DNA methylation, Infinium Methylation 450K array, Methylation EPIC array

## Abstract

**Background:**

Although epigenetic mechanisms are important risk factors for allergic disease, few studies have evaluated DNA methylation differences associated with atopic dermatitis (AD), and none has focused on AD with eczema herpeticum (ADEH+). We will determine how methylation varies in AD individuals with/without EH and associated traits. We modeled differences in genome-wide DNA methylation in whole blood cells from 90 ADEH+, 83 ADEH−, and 84 non-atopic, healthy control subjects, replicating in 36 ADEH+, 53 ADEH−, and 55 non-atopic healthy control subjects. We adjusted for cell-type composition in our models and used genome-wide and candidate-gene approaches.

**Results:**

We replicated one CpG which was significantly differentially methylated by severity, with suggestive replication at four others showing differential methylation by phenotype or severity. Not adjusting for eosinophil content, we identified 490 significantly differentially methylated CpGs (ADEH+ vs healthy controls, genome-wide). Many of these associated with severity measures, especially eosinophil count (431/490 sites).

**Conclusions:**

We identified a CpG in *IL4* associated with serum tIgE levels, supporting a role for Th2 immune mediating mechanisms in AD. Changes in eosinophil level, a measure of disease severity, are associated with methylation changes, providing a potential mechanism for phenotypic changes in immune response-related traits.

**Electronic supplementary material:**

The online version of this article (10.1186/s13148-019-0714-1) contains supplementary material, which is available to authorized users.

## Background

Atopic dermatitis (AD), a complex chronic skin disease, affects up to 30% of children. It often persists into adulthood [1, 2]. A primary symptom of AD is incessant pruritus [3] which typically has an intermittent course with flares and remissions. Eczema herpeticum (EH) is a rare but serious complication of AD. The primary predisposing factor for EH is HSV-1 exposure (in 2005–2010, 54% of persons in the USA aged 14–49 had HSV-1 infection [4]). In spite of HSV-1 infection being the primary environmental risk factor in the development of EH, only a small subset (less than 3%) of patients with AD have a history of EH (ADEH+). ADEH+ patients typically represent the severe end of the disease spectrum, with more severe skin disease. They have reduced interferon responses and are highly allergic with increased serum tIgE levels and eosinophilia [5–9]. Yet, the factors contributing to ADEH+ are unclear. To date, there is considerable evidence for genetic determinants associated with viral dissemination [10–12] and outcomes associated specifically with risk of ADEH+ and disease severity [6, 8, 9, 13–20].

DNA methylation, an epigenetic mechanism by which gene expression is regulated without alterations in nucleotide sequence, has been shown to contribute to the risk of complex diseases, notably autoimmune disorders and diseases of inflammation [21–24]. Risk of AD has been shown to be correlated with changes in genomic DNA methylation patterns [25–27] in lesional versus non-lesional AD skin [28]. However, no study has examined patterns of methylation specific to ADEH+. Common contributors to both ADEH− and ADEH+ are skin barrier abnormalities and immune dysregulation while risk factors for ADEH+ are early-onset and persistent severe AD [5, 29]. In spite of these known risks, the pathophysiology of ADEH+ still is not completely understood [30]. In addition to identifying a methylation-specific signature for ADEH+, a goal of the current study was to determine whether an easily accessible tissue such as whole blood can be the source of a biomarker which might facilitate the early diagnosis of patients with AD prone to disseminated viral infections, and whether or not methylation changes in the blood can distinguish between ADEH+ and ADEH−.

Using blood as a surrogate tissue to identify methylation changes associated with AD, and ADEH+ specifically, raises the challenge of performing an analysis that will control for signatures of immune response present in the blood. To account for differences in eosinophils across subjects, we estimated eosinophil fractions from the methylation data and included these fractions, along with those of other cell types composing each sample, in linear models. We contend that the CpGs associated with ADEH+ status and severity in this analysis should be independent of the specific immune response reflected by eosinophil levels.

However, similar to other recent reports focused on asthma and IgE-mediated allergic disease [31, 32], we found in further unadjusted analysis that the strongest factor to influence methylation differences was eosinophil count. To explore this phenomenon in our data, we also examined methylation differences without adjusting for eosinophil fractions in discovery subjects and found many sites that were different between ADEH+ and healthy control individuals and which were related to various disease severity measures. We present discovery and replication results for all analyses and conclude with a discussion of the challenges of interpreting results from blood methylation analysis for an immune-related disease like AD.

## Results

### Estimation of cell-type composition and assessment of model calibration

Estimated cell fractions were included in all models to ensure removal of confounding effects for either 7 or 6 cell types (neutrophils and eosinophils combined as granulocytes). See Additional file 1 for further details on models and plots to assess model calibration.

### Differentially methylated position (DMP) analysis: genome-wide dichotomous comparison

**Fig. 1:**
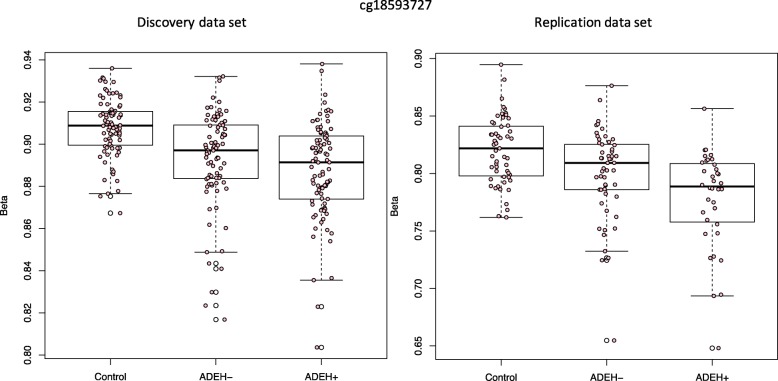
Methylation levels (% methylation) by group for cg18593727 for discovery (left) and replication (right) data sets.

Differential methylation analysis was performed for all pairwise comparisons of phenotype groups, adjusting for seven cell types, including eosinophils, estimated from the data: ADEH− against healthy controls, ADEH+ against healthy controls, and ADEH− against ADEH+ (see Table 1 for clinical characteristics of samples included in analysis). One CpG (cg18593727) showed genome-wide significantly differential methylation between the ADEH+ patients and the healthy control group in our discovery analysis and showed suggestive replication (discovery FDR adjusted *q*-value 0.0426, replication nominal *p* value 0.0345, Fig. 1). This CpG was annotated to the *HCLS1* gene (Hematopoietic Cell-Specific Lyn Substrate 1), a substrate of the antigen receptor-coupled tyrosine kinase, which plays a role in antigen receptor signaling for both clonal expansion and deletion in lymphoid cells.

### Differentially methylated position (DMP) analysis: targeted gene dichotomous comparison

**Fig. 2:**
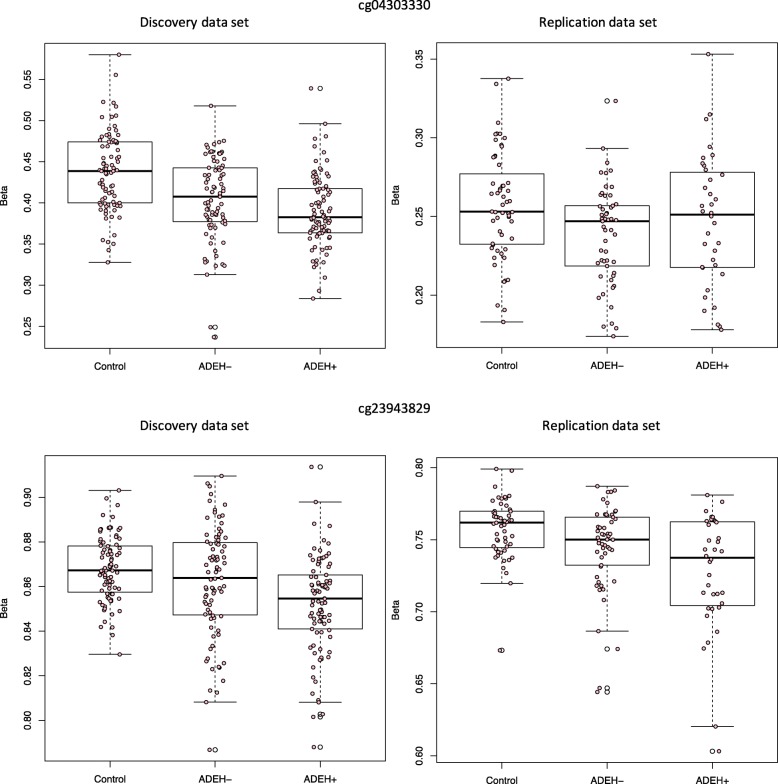
Box plots showing distribution of methylation levels (% methylation) by phenotype group for cg04303330 (top row) and cg23943829 (bottom row) for discovery (left) and replication (right).

Two CpGs, one in *IL4* (cg23943829) and one in *IL13* (cg04303330), showed significant differential methylation between ADEH+ and healthy controls in the discovery analysis (FDR adjusted *q*-values of 0.03 and 0.04, respectively) and suggestive significance, including similar effect sizes in the same direction, in replication (nominal *p* values of 0.051 and 0.094, respectively, Table 2, Fig. 2, Additional file 2: Table S2).

### DMP analysis: serum tIgE levels in *IL4*, *IL13*, and *IL4R*

**Fig. 3:**
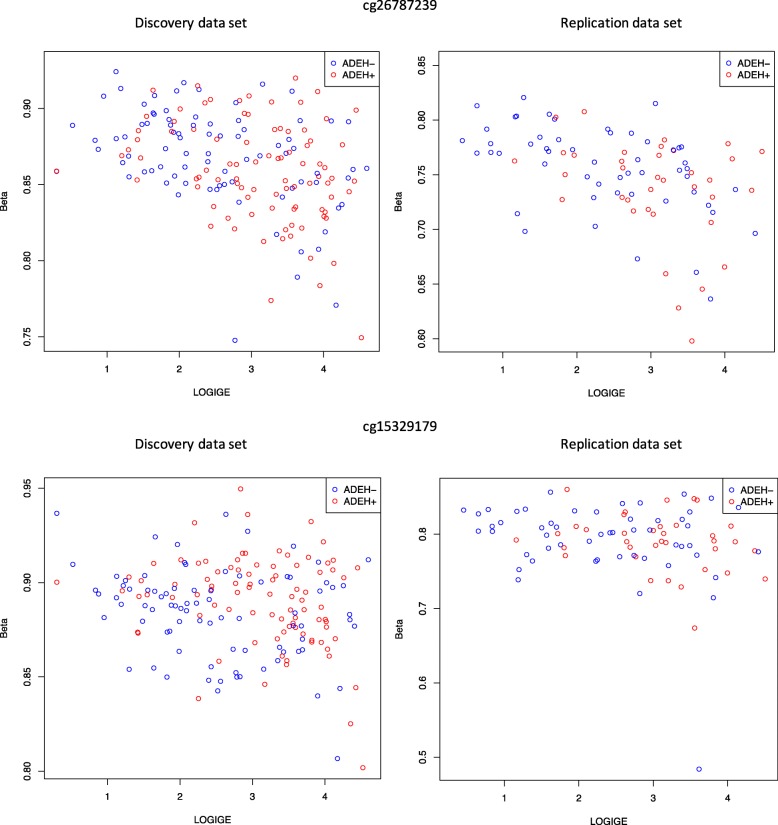
Scatter plots showing correlation between methylation levels (% methylation, Beta) and log serum tIgE levels for cg26787239 (top row) and cg15329179 (bottom row) for discovery (left) and replication (right).

To address a previous link between *IL4*, *IL13*, and *IL4R* and serum tIgE levels [33–35], we performed a severity analysis limited to the 26 CpGs in these three regions. We found six CpGs in the discovery analysis, one of which replicated after Bonferroni correction for the 9 tests carried out in the replication analysis (cg26787239 in *IL4*, discovery FDR adjusted *q*-value 0.0042, replication *p* value 0.0045) and one with suggestive replication (cg15329179 in *IL13*, discovery FDR adjusted *q*-value 0.0042, replication *p* value 0.016), both showing association between serum tIgE and methylation levels (Table 3, Fig. 3, Additional file 2: Table S3).

### Differentially methylated position (DMP) analysis: dichotomous comparisons with six cell types

Based on results from recent work investigating methylation patterns in blood in atopy-related disease (e.g., asthma, Arathimos et al. [31], Wei Chen et al. [32]), we investigated differential methylation patterns without adjusting for differences in eosinophil fraction between subjects. Differential methylation analysis was performed for all pairwise comparisons of phenotype groups, adjusting for the six main cell types estimated from the data: ADEH− against healthy controls, ADEH+ against healthy controls, and ADEH− against ADEH+. Comparing ADEH+ with healthy controls, 490 CpGs were differentially methylated (FDR-adjusted *q*-value < 0.05) and comparing ADEH− with Controls, six CpGs were differentially methylated (FDR-adjusted *q*-value < 0.05) (Additional file 3: Table S4), of which five were also in the group of ADEH+ to healthy control DMPs. Interestingly, there were no CpGs that were significantly different between the ADEH− and ADEH+ phenotype groups, although mean methylation among ADEH− individuals at CpGs with significant ADEH+ to healthy control differences was to a large extent intermediate (452 of 490 CpGs, 92.2%) between ADEH+ and healthy controls’ methylation (Additional file 1: Figure S5).

### DMP analysis: severity measures with six cell types

To further investigate the trend whereby ADEH− individuals have methylation values intermediate to healthy controls and ADEH+ individuals but show no statistically significant differences when compared to the ADEH+ patients, we performed an analysis investigating the relationship between different AD severity measures and methylation at the 491 CpGs that were significant (FDR adjusted *q*-values < 0.05) for at least one pairwise comparison. Severity measures included serum tIgE levels, eosinophil counts [36–38], EASI score, and Rajka-Langeland score; analyses were performed within the collective AD group (ADEH−, ADEH+; *N* = 173) for each of these sub-phenotypes.

The strongest associations were observed for eosinophil counts with 431 out of 491 sites showing significant association (FDR-adjusted *q*-values < 0.05) between methylation and eosinophil counts (Additional file 4: Tables S5–S8, Additional file 1: Figure S6). A significant association was observed for serum tIgE levels at 335 sites, for EASI scores at 337 sites, and Rajka-Langeland scores at 276 sites.

### Gene ontology analysis for results from analysis with six cell types

Gene ontology (GO) analysis was performed on the 490 CpGs with significant association from the analysis adjusting for six cell types comparing ADEH+ to healthy controls. Two GO terms were enriched (FDR-adjusted *q*-value < 0.1) in the ADEH+ versus healthy controls group. Both terms, GO:0002761 and GO:0002573, are biological processes involved in the regulation of myeloid leukocyte differentiation (Additional file 5: Table S9).

## Discussion

With adjustment for the full set of seven cell types in the model, one CpG significant in the discovery phase showed suggestive replicated association with the ADEH+ phenotype compared to healthy controls in a genome-wide test (Fig. 1). This CpG is located 1 kb upstream of the *HCLS1* gene (Hematopoietic Cell-Specific Lyn Substrate 1), encoding a substrate of the antigen receptor-coupled tyrosine kinase, which plays a role in antigen receptor signaling for both clonal expansion and deletion in lymphoid cells. One of its related pathways includes the Immune response FcεRI pathway and FcεRI-mediated dendritic cell signaling and antigen presentation, which promotes the development and activation of Th2 cells and contributes to allergic inflammatory diseases [39], suggesting a potential role in allergic disease.

Given extensive prior work conducted on the genetics of AD, we focused our attention on a specific subset of candidate genes for AD. Using a set of 129 CpGs that belong to AD candidate genes of interest (*FLG* [5, 17, 18, 25, 29, 40], *LCE1B* [40, 41], *RPTN* [41, 42], *IL4*, *IL13* [18, 30, 33–35, 40] and its receptors, *IFNs* [6, 7, 10] and *TSLP* [13, 14, 18, 30, 40], see Additional file 1 Table S10), we identified CpGs significant by phenotype in the discovery and suggestive in the replication data sets in the *IL13* and *IL4* genes (Fig. 2, Table 2). This suggests there is a significant association between methylation and phenotype in these genes, even when taking differences in eosinophils into account. We also found replicated association between methylation and serum tIgE levels for a CpG in the *IL4* gene region and suggestive replication for a CpG in the *IL13* gene region (Fig. 3, Table 3). The CpG in the *IL4* gene region, cg26787239, which was significantly associated with serum tIgE levels, was also the most significant signal in recent work performed in an asthma cohort [43].

**Table 1:**
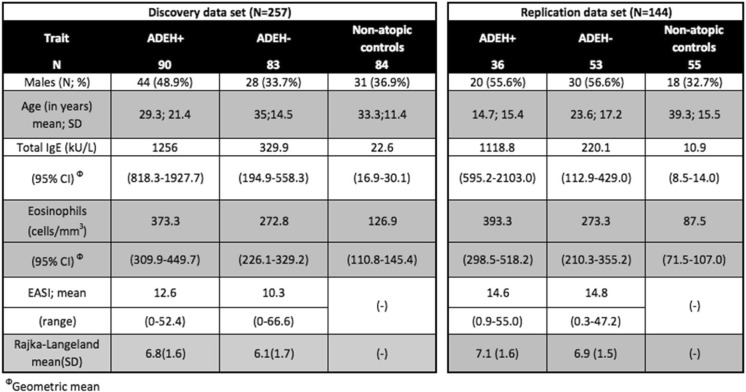
Clinical characteristics table for samples analyzed in discovery and replication data sets

**Table 2:** Summary statistics from discovery and replication from gene-based analysis comparing ADEH+ individuals to non-atopic healthy controls, adjusted for Eos and Neu fractions. Both significant CpGs from the discovery stage were suggestive for replication (based on a Bonferroni correction for 9 tests)

				Discovery data set			Replication data set	
ID	CHR	POS (hg19)	GENE	Diff_EHNA	Pval_EHNA	qval_EHNA	Diff_EHNA	Pval_EHNA
cg23943829	5	132009111	*IL4*	− 0.1425	0.0002	0.0287	− 0.0996	0.0509
cg04303330	5	131992430	*IL13*	− 0.1516	0.0007	0.044	− 0.1226	0.0941

*Diff*_*EHNA* difference in methylation values (*M*-value scale) between ADEH+ and healthy control group, *Pval*_*EHNA p* value obtained from ADEH+ vs healthy control differential methylation analysis, *qval*_*EHNA* FDR corrected *q* values calculated on a set of CpGs in our genes of interest

**Table 3:** Summary statistics from discovery and replication from gene-based analysis for serum tIgE levels, not adjusted for Eos and Neu fractions. One significant CpG from the discovery stage replicated (based on a Bonferroni correction for 9 tests) with an additional CpG suggestive of replication

				Discovery data set			Replication data set	
ID	CHR	POS (hg19)	GENE	Diff_IgE_cases	Pval_IgE_cases	qval_IgE_cases	Diff_IgE_cases	Pval_IgE_cases
cg26787239	5	132008525	*IL4*	− 0.1073	0.0002	0.0042	− 0.07098	0.0045
cg15329179	5	131993728	*IL13*	− 0.08296	0.00033	0.0042	− 0.0628	0.01595
cg06641959	16	27325254	*IL4R*	0.05223	0.00251	0.0163	− 0.0035	0.8569
cg06967316	5	131993853	*IL13*	− 0.04903	0.00192	0.0163	− 0.0096	0.6556
cg14523284	5	131993614	*IL13*	− 0.06025	0.00335	0.0174	0.0049	0.7816
cg23943829	5	132009111	*IL4*	− 0.0549	0.0055	0.0237	− 0.0258	0.2095

*Diff*_*IgE*_*cases* difference in methylation values (M-value scale), *Pval*_*IgE*_*cases p* value obtained from differential methylation analysis for serum tIgE levels, *qval*_*IgE*_*cases* FDR corrected q values calculated on set of CpGs in *IL4* and *IL13* genes

Reviewing published work examining the association between changes in epigenetics and AD, we found no prior study that accounted for differences in eosinophil levels, so we used our 491 methylation changes associated with phenotype without adjusting for eosinophils for comparison. Compared to Everson et al. [44], which focused on both case-control differences in AD and a comparison of individuals with low or high IgE levels, 11 of our CpGs overlapped with the top 22 CpGs significantly different between individuals with AD compared to controls [44]. However, we found no overlap with the top 140 CpGs presented in [45], which were the result of a related study that used random forests to compare individuals with and without eczema. Reasons for this lack of overlap include methodological differences and differences in the phenotype being considered.

In addition to AD status, we also considered measures of AD severity, specifically serum tIgE levels, which has previously been associated with AD severity [36–38]. The selection of the *IL4*, *IL13*, and *IL4R* regions for investigation of serum tIgE levels was in fact based on prior work showing that suppression of these Th2 cytokines decreases serum tIgE levels [34], that these genes are involved in the pathway influencing cytokine- and receptor-mediated regulation of IgE [35], and that they are implicated in genetic association studies examining serum tIgE levels as an outcome [33]. In addition, genetic loci near *IL13* were shown to be methyl-QTLs for CpGs in the promoter of *IL13* indicating that methylation could play a functional role, linking genetics to outcome [46]. In this context, our results linking methylation near *IL13* and *IL4* to serum tIgE levels implicate a role for methylation in determining the presence of serum tIgE or vice versa.

Recent work has shown that a major contributor to observed differences in methylation from whole blood between groups with different allergic disease phenotypes is a cross-sample variation in cell type composition, in particular, the eosinophil fraction [24, 31, 32]. Specific to AD, prior work has shown that the peripheral eosinophil counts or serum levels of eosinophil-derived proteins are a measure of disease severity [36–38]. To explore this, we modeled the outcome between methylation and phenotype without adjusting for the estimated fraction of eosinophils in each sample to determine whether differences in eosinophil fractions might contribute directly to differences in methylation that then affect the outcome of interest. This analysis with adjustment for six default cell types (eosinophils and neutrophils combined as granulocytes) identifies biologically relevant signals strongly associated with different diseases from previous studies [40, 47]. The two gene ontology (GO) terms associated with these 490 CpGs were both biological processes involved in the regulation of myeloid leukocyte differentiation. While we were not able to find specific links between myeloid leukocyte differentiation and AD, we identified at least one reference discussing the role of myeloid dendritic cells in AD [48]. Interestingly, from the severity analyses targeting serum tIgE levels, eosinophil counts, EASI score and Rajka-Langeland scores, eosinophil counts showed the largest number of CpGs that correlated with methylation levels. Given the strong evidence that ADEH+ patients have higher serum tIgE levels and circulating eosinophil counts compared to ADEH− patients [5, 36–38], it is of interest that we found methylation changes associated with both of these phenotypes.

Other phenotypes related to allergic response, such as asthma, face similar challenges with cell composition confounding. In fact, methylation analysis recently performed in asthma patients identified CpGs strongly associated with asthma that were also significant in our analyses, in both cases without adjustment for eosinophil fractions. Specifically, cg10159529 (*IL5RA*) and cg27469152 in the 3′UTR region of the *EPX* gene were highly significant in the asthma analysis [31] and in our comparisons of ADEH+ patients versus healthy controls (FDR-adjusted *q*-value < 0.05) when not adjusting for eosinophils. These genes have known links to both eosinophil functions and allergic sensitization. DNA methylation in CpGs annotated to the *ZFPM1* (cg04983687, cg08940169) and *ACOT7* (cg09249800, cg21220721, cg11699125) genes were also shown to be strongly associated in the asthma study and in our analysis focusing on ADEH+. However, as in our AD study, if eosinophil fractions were adjusted for in the asthma analysis, residual differential methylation comparing asthma cases to controls was nearly absent. While there is strong confounding between differences in eosinophil count, differences in methylation and AD status, these changes in methylation could lead to functional differences that are a consequence of this change in cellular composition.

## Conclusions

In summary, we find replication for one CpG associated with serum tIgE in the *IL4* gene and suggestive replication for four CpGs associated with EH compared to healthy controls or with AD severity measures, three of which fall in two genes of interest, *IL13* and *IL4*. As previous gene expression studies have identified higher expression of IL4 and IL13 in a specific disease group and have led to a treatment using a monoclonal antibody (dupilumab) to block these molecules [34], our hope is that a similar treatment could arise from the results of this study, in conjunction with further work on the epigenetics of eczema herpeticum. We also show that eosinophil level plays a significant role in methylation patterns in individuals with AD, presenting both a potential confounder and a potential mechanism for enacting methylation changes that could lead to phenotypic changes.

## Methods

### Study Subjects

#### Discovery

ADEH+ subjects were defined as patients with AD who had at least one previous EH episode as physician documented in Beck et al. [5]. ADEH− subjects were defined as patients with AD with no history of EH. Non-atopic subjects were defined as having no individual or family history of atopy and average total IgE less than 100 kU/L, as in [5]. Methylation studies were conducted on a subset of participants including 100 ADEH+, 100 ADEH−, and 100 non-atopic subjects (NA), see Additional file 1: Table S1.

#### Replication

In order to replicate our initial findings, methylation studies were conducted on 56 ADEH+, 56 ADEH−, and 56 non-atopic subjects (NA), see Additional file 1: Table S1**.**

### Measurements of AD severity

All study participants underwent a detailed history, physical examination, disease severity assessment, and blood draw. For all AD patients, disease severity was assessed by the Rajka-Langeland and the Eczema Area and Severity Index (EASI) scoring systems. The total eosinophil count (cells/mm3) was calculated from the “CBC with differential” blood test. Log-transformed values for IgE, Eosinophil count, EASI, and phadiatop were used for the analysis. In order to adjust for any values less than 1 in the data set, before applying a log_10_ transformation, we added 10 to all eosinophil counts and 1 to all EASI and phadiatop values. A Box-Cox transformation with a lambda of 1.5 was applied to the Rajka-Langeland score in order to normalize the distribution.

### Quality control and preprocessing

#### Sample preparation

Standard protocols for preparing DNA methylation data using Illumina’s array technology were followed for discovery and replication. See Additional file 1 for further details.

#### Discovery data set

Methylation data was quality control tested using the “minfi” R-package [49]. All analyses throughout, with one exception below, were run using R version 3.3.1 and minfi 1.18.6. A total of 39 samples (15 Controls, 15 ADEH− and 9 ADEH+ samples) were removed from further analysis as a result of either low methylated/unmethylated median values (Additional file 1: Figure S1) (12 controls, 14 ADEH−, and 7 ADEH+ samples) or due to gender mismatches (4 controls: 1 also failed QC, 1 ADEH−, and 2 ADEH+ samples) between the phenotype annotations and the calls generated by the minfi *getSex* function (Additional file 1: Figure S2). Three samples (2 Controls and 1 ADEH+ sample) were excluded from the study as they did not meet the requirement of “Non-Hispanic” and were ineligible per the protocol requirements. One of the three samples (Control) was also a QC failure so there were 41 samples excluded. Samples were run in seven batches and excluding 41 samples left the last batch with just two samples (ADEH−). In order to balance the samples within batches on their phenotype, these two samples were also excluded. After filtering samples for quality control (see Additional file 1), the remaining 257 samples (Table 1) were normalized using the minfi stratified quantile option. Probes mapping either to the X or Y chromosomes or in close proximity to SNPs (at the CpG site or in the single-base extension site for the array probe) were removed, leaving 456,513 CpG probes for analysis.

#### Replication data set

Because the Methylation 450K array was discontinued by Illumina between generation of the discovery data set and the replication data set, the Methylation EPIC chip with 866,836 probes was used for our replication samples. Of the CpGs interrogated by the Methylation 450K array, 93% were also included on the EPIC 850K chip. The CpGs moved forward to replication from our discovery data set were among those included on the new platform.

Methylation data was quality control tested using the minfi R-package [49]. Of the 168 samples run, there was one sample that failed the initial experimental QC. One hundred and sixty-seven samples were run through the QC pipeline. Including that sample, a total of 6 samples (1 Control, 3 ADEH−, and 2 ADEH+ samples) were removed from further analysis as a result of either technical issues while performing the assay or low methylated/unmethylated median values (Additional file 1: Figure S1**)** (1 Control, 1 ADEH−, and 1 ADEH+ samples) or due to gender mismatches (2 ADEH−: 1 also failed QC and 2 ADEH+ samples) between the phenotype annotations and the calls generated by the minfi *getSex* function (Additional file 1: Figure S2). There were 19 samples that were inadvertently included in discovery and replication data sets. These 18 samples (ADEH+) were excluded (1 was a QC failure) from the analysis. After filtering samples for quality control (see Additional file 1), the remaining 144 samples (Table 1) were normalized using the minfi stratified quantile option. Probes mapping either to the X or Y chromosomes or in close proximity to SNPs (at the CpG site or in the single-base extension site for the array probe) were removed, leaving 817,465 CpG probes for analysis. See Additional file 1 for further details about QC.

### Data analysis

The following will provide a brief overview of our data analysis and modeling pipeline. Additional details are available in Additional file 1.

#### Cell type distribution

**Fig. 4:**
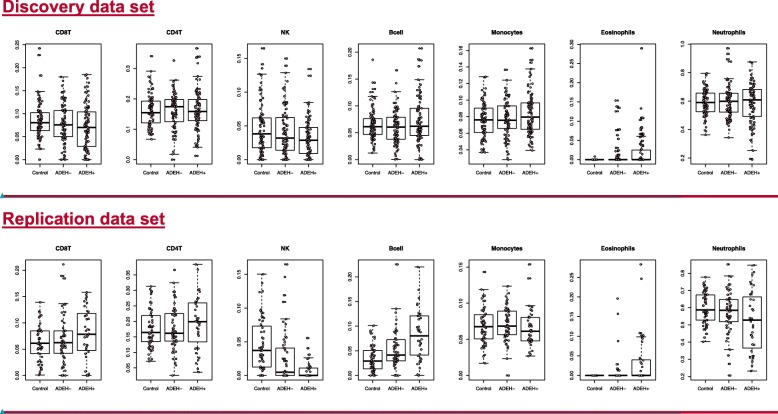
Box plots for all cell composition estimates for clean samples by phenotype groups for seven cell types for the discovery data set (top row) and the replication data set (bottom row).

Significant heterogeneity among different cell types as found in blood (either whole or in PBMC fractions) has been conclusively demonstrated to confound differential methylation studies [50]. To test and control for the possibility of apparent changes in methylation status that may result from changes in cell-type composition across group, we used the minfi R-package, which incorporates the method of Jaffe and Irizarry [50] to predict blood cell count distributions for up to seven cell types: CD4T, CD8T, eosinophils, neutrophils, B-cells, natural killer cells, and monocytes. Boxplots of estimated cell fractions of each of seven cell types for individuals split by phenotype group are shown in Fig. 4. We included these seven components in our initial models. In addition, a combined granulocyte signal composed of eosinophil and neutrophil components can be estimated, resulting in six cell-type fractions. We used these six cell-type estimates in later analyses.

#### Batch adjustment

The missMethyl package was developed specifically for analysis of 450K and EPIC array data and offers an implementation of RUV-inverse [51] called RUVm [52] as a solution for removing batch effects and unknown, unwanted variation from the data. We used this method to ensure we were appropriately adjusting for batch effects in our linear models.

#### Detection of differentially methylated positions (DMPs)

In both the discovery and replication analyses, DMPs were detected using standard linear modeling approaches with covariates age, sex and batch, and estimates of either six or seven cell-type fractions, along with batch effect factors (estimated by RUVm). More specifically, our model was of the form:

$$ {\displaystyle \begin{array}{l}\mathrm{DNAm}\left(M-\mathrm{value}\right)\sim {\beta}_0+{\beta}_1\Big(\mathrm{Predictor}\ \mathrm{of}\\ {}\mathrm{interest}\Big)+{\beta}_2\left(\mathrm{Sex}\right)+{\beta}_3\left(\mathrm{Age}\right)+{\beta}_4\left(\mathrm{CD}8\mathrm{T}\right)+{\beta}_5\left(\mathrm{CD}4\mathrm{T}\right)+{\beta}_6\left(\mathrm{NK}\right)+{\beta}_7\left(\mathrm{B}\;\mathrm{cell}\right)+{\beta}_8\left(\mathrm{monocytes}\right)+{\beta}_9\left(\mathrm{eosinophils}\right)+{\beta}_{10}\left(\mathrm{neutrophils}\right)+{\sum}_{l=1}^L{\gamma}_l{C}_l\end{array}} $$

where the term ${\sum}_{l=1}^L{\gamma}_l{C}_l$ includes factors estimated to control for batch effects, and *M*-value refers to logit-transformed percent methylation values.

*P*-value distribution adjustment was performed using bacon [53] and then FDR-adjusted *q*-values were estimated. In all cases, an FDR cutoff of 0.05 was used to declare significance in discovery; for the 9 results carried forward to replication, a Bonferroni adjustment was used, giving a *p* value cutoff of 0.0056. For further details of modeling choices, please see Additional file 1. The following analyses were carried out:

1. Genome-wide tests of differential methylation by group (ADEH+ vs healthy control, ADEH− vs healthy control, ADEH+ vs ADEH−) adjusting for 7 cell types**.**
2. Gene-based tests of differential methylation by group (ADEH+ vs healthy control, ADEH− vs healthy control, ADEH+ vs ADEH−), adjusting for 7 cell types**.** A set of 129 CpGs that mapped to a set of genes previously shown to be of interest in EH, specifically *FLG*, *LCE1B*, *RPTN*, *IL4*, *IL13* and its receptors, *IFNs*, and *TSLP* was considered.
3. Genome-wide tests of differential methylation by group (ADEH+ vs healthy control, ADEH− vs healthy control, ADEH+ vs ADEH−) adjusting for 6 cell types (granulocytes instead of eosinophils and neutrophils). For all CpGs significant from this analysis, further modeling was performed using the discovery data to assess the association between severity measures (eosinophil counts, total serum IgE (tIgE) levels, EASI score, and Rajka-Langeland score) and methylation. Results from this analysis were declared significant if *q* < 0.05. No results from this analysis were carried forward for replication.
4. Gene-based tests of differential methylation by serum tIgE level, adjusting for 6 cell types (granulocytes instead of eosinophils and neutrophils). A set of 26 CpGs in the *IL4*, *IL13*, and *IL4R* genes was considered to test a specific hypothesis of the role of these genes on serum tIgE.

#### Gene ontology (GO) enrichment analysis

Gene ontology enrichment analysis was performed with the *gometh* function in missMethyl, which is designed specifically to address potential biases in measuring gene-set enrichment with the 450K methylation array [54].

## Additional files

Additional file 1: Supplementary text. **Figure S1**. QC plot of methylated to unmethylated median intensities for discovery and replication data sets. **Figure S2**. Plot of chrX vs chrY median intensities to identify gender mismatches for discovery and replication data sets. **Figure S3**. QQ plots of *p* values (three phenotype comparisons) from models with seven cell types (top) and six cell types (bottom) prior and post bacon adjustment. **Figure S4**. QQ plots of *p* values (severity analysis) from models with seven cell types (top) and six cell types (bottom) prior and post bacon adjustment. **Figure S5**. Box plots for top 27 CpGs significant in ADEH+ vs controls analysis. **Figure S6**. Scatter plots of top 27 CpGs significant in eosinophil-methylation analysis showing eosinophil levels against methylation values. **Table S1**. Clinical Characteristics table for all samples in discovery and replication data sets. **Table S10**. References to support the selection of genes for our gene-based analysis. (DOCX 4040 kb)

Additional file 2: **Table S2** and **S3**. DMPs significant from gene-based analysis for both phenotype groups and severity scores. (XLSX 31 kb)

Additional file 3: **Table S4**. DMPs significant from ADEH− vs controls and/or ADEH+ vs controls analysis at an FDR threshold of 0.05 from model adjusting for six cell types. (XLSX 91 kb)

Additional file 4: **Tables S5–S8.** DMPs significant from severity analysis to follow up on results in Additional file 3: Table S4. (XLSX 120 kb)

Additional file 5: **Table S9**. Gene ontology (GO) analysis results for ADEH+ vs healthy controls. (XLSX 11 kb)

## Data Availability

The data sets generated and/or analyzed during the current study are not made publicly available due to data security requirements.

## Abbreviations

AD: Atopic dermatitis
ADEH−: Atopic dermatitis without a history of EH
ADEH+: Atopic dermatitis with a history of EH
DMP: Differentially methylated probe
EASI: Eczema Area and Severity Index
EH: Eczema herpeticum
FDR: False discovery rate
NA: Non-atopic
