# Supplementary material for: Replicated methylation changes associated with eczema herpeticum and allergic response
Source: Clin Epigenetics. 2019 Aug 23;11:122. doi: 10.1186/s13148-019-0714-1 (PMC6706929; doi:10.1186/s13148-019-0714-1)
Supplement: Supplementary file 1 — Supplementary text. Figure S1. QC plot of methylated to unmethylated median intensities for discovery and replication data sets. Figure S2. Plot of chrX vs chrY median intensities to identify gender mismatches for discovery and replication data sets. Figure S3. QQ plots of p values (three phenotype comparisons) from models with seven cell types (top) and six cell types (bottom) prior and post bacon adjustment. Figure S4. QQ plots of p values (severity analysis) from models with seven cell types (top) and six cell types (bottom) prior and post bacon adjustment. Figure S5. Box plots for top 27 CpGs significant in ADEH+ vs controls analysis. Figure S6. Scatter plots of top 27 CpGs significant in eosinophil-methylation analysis showing eosinophil levels against methylation values. Table S1. Clinical Characteristics table for all samples in discovery and replication data sets. Table S10. References to support the selection of genes for our gene-based analysis. (DOCX 4040 kb) [file 13148_2019_714_MOESM1_ESM.docx]

**Supplementary Online Content**

**Supplementary Methods**

**Supplementary Figure S1.** QC plot of methylated to unmethylated median intensities for discovery and replication data sets.

**Supplementary Figure S2.** Plot of chrX vs chrY median intensities to identify gender mismatches for discovery and replication data sets.

**Supplementary Figure S3.** QQ plots of p-values (three phenotype comparisons) from models with seven cell types (top) and six cell types (bottom) prior and post bacon adjustment

**Supplementary Figure S4.** QQ plots of p-values (severity analysis) from models with seven cell types (top) and six cell types (bottom) prior and post bacon adjustment

**Supplementary Figure S5.** Box plots for top 27 CpG’s significant in ADEH+ Vs controls analysis

**Supplementary Figure S6.** Scatter plots of top 27 CpGs significant in eosinophil-methylation analysis showing eosinophil levels against methylation values.

**Supplementary Table S1.** Clinical Characteristics table (discovery N=297, replication N=167)

**Supplementary Tables S2 and S3.** DMPs significant from gene based analysis for both phenotype groups and

severity scores: **Additional file 2.xlsx**

**Supplementary Table S4.** DMPs significant from ADEH- Vs controls and/or ADEH+ Vs controls analysis at an FDR threshold of 0.05 from model adjusting for six cell types: **Additional file 3.xlsx**

**Supplementary Tables S5-S8**. DMPs significant from severity analysis to follow up on results in Table 4: **Additional file 4.xlsx**

**Supplementary Table S9.** Gene Ontology (GO) analysis results for ADEH+ vs healthy controls: **Additional file 5.xlsx**

**Supplementary Table S10.** References to support the selection of genes for our gene based analysis.

**Supplementary Methods**

***Sample Processing***

***Isolation of Whole Blood:*** Venous blood was collected by simple venipuncture under aseptic conditions.

***Purification of DNA***: Genomic DNA was isolated from whole blood using the MagAttract DNA Mini M48 Kit on the automated BioRobot M48 workstation (Qiagen).

***Bisulfite Treatment***: Isolated DNA was bisulfite converted using the EZ DNA Methylation Gold kit (Zymo, Irvine, CA). The product of this process contains cytosine converted to uracil if it was previously unmethylated.

***Genomic DNA Amplification***: The bisulfite treated DNA was subjected to whole genome amplification (WGA) via random hexamer priming and Phi29 DNA polymerase, and the amplification products were then enzymatically fragmented (1), purified from dNTPs, primers, and enzymes, and applied to the Illumina chip (2).

***Hybridization and Single-base Extension:*** The bisulfite converted amplified DNA products were denatured into single strands and hybridized to the Infinium HumanMethylation450 BeadChip via allele specific annealing to either the methylation specific probe or the non-methylation probe. The Infinium HumanMethylation450 BeadChip contains more than 485,000 methylation sites per sample at single-nucleotide resolution. It covers 99% of RefSeq genes, with an average of 17 CpG sites per gene region distributed across the promoter, 5'UTR, first exon, gene body, and 3'UTR. It covers 96% of known CpG islands as well as many CpG sites outside of CpG islands. Hybridization to the chip was followed by single base extension with labeled di-deoxynucleotides.

***Fluorescence Staining and Scanning of Chip***: The hybridized Beadchip was stained, washed, and scanned to show the intensities of the un-methylated and methylated bead types using Illumina’s Iscan System. The raw data was analyzed by the scanner software (Genome Studio), and the fluorescence intensity ratios between the two bead types were calculated, referred to as Beta values. A Beta value of 0 is equivalent to non-methylation of the locus; a value of 1 equals to total methylation; a value of 0.5 means that on average one of the two chromosomal copies was methylated and the other was not (1). Logit transformed beta values, called M values, are shown to perform much better in terms of Detection Rate (DR) and True Positive Rate (TPR) for both highly methylated and unmethylated CpG sites (3). Hence, M values were used for all our statistical modeling and analysis.

***Measurements of AD severity:***

EASI is a standardized grading system (range of score, 0-72) that assesses erythema, excoriation, lichenification, infiltration and/or population (4). The Rajka-Langeland score (RLS) rates extent, course, and itch intensity separately and yields a score from 0-9 (5). The RLS system provides a broad and somewhat historical view of a subject's AD severity, whereas the EASI provides a more sensitive measure of disease severity at the time of enrollment. Both EASI and RLS are available for AD patients only and are not measured in controls. For all study participants, blood samples were sent to Quest Diagnostics Laboratory for a complete blood count (CBC) with differential and to the Dermatology, Allergy and Clinical Immunology Laboratory (DACI) at Johns Hopkins Asthma and Allergy Center, for total serum IgE (tIgE) levels. Using the UniCap 250 system (Pharmacia and Upjohn), the DACI laboratory performed the tIgE (kU/L) tests on serum samples from all ADEH+ and ADEH- subjects.

***Quality Control and preprocessing:***

Discovery data set:

A total of 39 samples (15 Controls, 15 ADEH- and 9 ADEH+ samples) were removed from further analysis as a result of either low methylated/unmethylated median values (**Supplementary Figure S1**) (12 controls, 14 ADEH- and 7 ADEH+ samples) or due to gender mismatches (4 controls: 1 also failed QC, 1 ADEH- and 2 ADEH+ samples) between the phenotype annotations and the calls generated by the ‘minfi’ *getSex* function (**Supplementary Figure S2**). Three samples (2 Controls and 1 ADEH+ sample) were excluded from the study as they did not meet the requirement of “Non-Hispanic” and were ineligible per the protocol requirements. One of the three samples (Control) was also a QC failure so there were 41 samples excluded. Samples were run in seven batches and excluding 41 samples left the last batch with just two samples (ADEH-). In order to balance the samples within batches on their phenotype, these two samples were also excluded.

*Replication data set:*

Of the 168 samples run, there was one sample that failed the initial experimental QC. 167 samples were run through the QC pipeline. Including that sample, a total of 6 samples (1 Control, 3 ADEH- and 2 ADEH+ samples) were removed from further analysis as a result of either technical issues while performing the assay or low methylated/unmethylated median values (**Supplementary Figure S1)** (1 Control, 1 ADEH- and 1 ADEH+ samples) or due to gender mismatches (2 ADEH-: 1 also failed QC and 2 ADEH+ samples) between the phenotype annotations and the calls generated by the minfi *getSex* function (**Supplementary Figure S2)**. There were 19 samples that were inadvertently included in discovery and replication data sets. These 18 samples (ADEH+) were excluded (1 was a QC failure) from the analysis.

***Results:***

***Estimation of cell-type composition and assessment of model calibration***Boxplots of estimated cell fractions of each of seven cell types (CD4T, CD8T, monocytes, eosinophils, neutrophils, natural killer cells and B-cells) for individuals split by phenotype group are shown in **main text** **Figure 4**. Some significant or suggestive differences were observed between groups (CD8T cells: ADEH+ vs controls p=0.03, NK cells: ADEH- vs controls p=0.002, ADEH- vs ADEH+ p=0.04, Monocytes: ADEH+ vs controls p=0.018, eosinophils: ADEH- vs controls p=0.002, ADEH+ vs controls p=2.98e-05, two-sample t-test; all other p-values > 0.05).; however, estimated cell fractions were included in all further models to ensure removal of confounding effects for these cell types. In addition to these seven components, additional modeling was performed combining neutrophils and eosinophils into granulocytes. Few significant or suggestive differences were observed between groups (natural killer cells: ADEH+ vs controls p=0.0008, ADEH- vs ADEH+ p=0.025, B-cells: ADEH- vs ADEH+ p=0.051, two-sample t-test; all other p-values > 0.05)

To assess model calibration, quantile-quantile plots were made pre- and post-distributional adjustment with *bacon* for the three phenotype comparisons and also severity analysis (**Supplementary Figures S3 and S4**), with post-adjustment results showing little evidence of overall test-statistic inflation (inflation factor lambda ADEH- vs healthy controls: 1.012, ADEH+ vs healthy controls: 1.014, ADEH- vs ADEH+: 1.017).

***Data analysis***

***Cell Type Distribution***

Significant heterogeneity among different cell types as found in blood (either whole or in PBMC fractions) has been conclusively demonstrated to confound differential methylation studies where apparent changes in methylation status may result more from changes in cell type distributions than from methylation level changes within a particular cell type (6). In order to test and control for this possibility, a function in the minfi R-package which incorporates the method of Jaffe and Irizarry (6) to predict blood cell count distributions was used. Purified (flow sorted) subsets from granulocytes, lymphocytes, and monocytes were used to identify a subset of cell-type specific CpGs which can be used to accurately predict cell type distributions in Illumina 450K methylation samples. This method as invoked by the *estimateCellCount* function in minfi (7) is a useful and significant advance on previous population-based methods (8). Cell type distribution was estimated by first generating the cell type coefficients (i.e. proportions) for each sample in the dataset. The function *estimateCellCount* provides estimated fractions for up to seven cell types: CD4T, CD8T, eosinophils, neutrophils, B-cells, natural killer cells and monocytes. These are the seven components we included in our initial models, following common practice. In addition, a combined granulocyte signal composed of eosinophil and neutrophil components can be estimated. We used these estimates in later analyses. Since cell estimates could not be extracted for the EPIC data set using older versions of minfi, *minfi 1.20.2* was used only to extract the cell estimates for replication data set.

***Batch adjustment***

The missMethyl package was developed specifically for analysis of 450K data and offers an implementation of RUV-inverse (9) called RUVm (10) as a solution for removing batch effects and unknown, unwanted variation from the data. This unwanted variation may be due to processing batches or other unmeasured confounders, such as temperature or humidity on the day that samples were processed together. To accurately estimate the components of unwanted variation, the two-stage RUVm method relies on negative control probes that are assumed not to be associated with the biological factor of interest. First, a differential methylation analysis using the 613 Illumina negative control probes with RUV-inverse is performed. Based on results from this first analysis, empirical controls, which are not associated with the outcome of interest in the first round of modeling, are identified. The empirical controls are then used by RUV-inverse in the second stage of analysis.

The *RUVfit* function runs this method and takes as input a matrix of M-values, a design matrix, the coefficient to be tested and a vector indicating the initial negative control probes. The *RUVadj* function adjusts the variance estimates using empirical Bayes shrinkage (probably unnecessary here due to large sample sizes) and the *topRUV* function is used to extract the top significantly differentially methylated CpG sites, after adjusting for the unwanted variation.

***Detection of differentially methylated positions (DMPs) by group***

We fit a linear model on the M-value scale. Covariates included age, sex and batch, as well as estimates of seven cell-type fractions (to control for potential confounding by outcome associated cell-types such as eosinophils) and batch effect factors (estimated by RUVm). More specifically our model was of the form:

*DNAm(M-value) ~* β*_0_+*β*_1_(Predictor of interest)+*β*_2_(Sex)+*β*_3_(Age)+*β*_4_(CD8T)+*β*_5_(CD4T)+*β*_6_(NK)+*β*_7_(B cell)+*β*_8_(monocytes)+*β*_9_(eosinophils) +*β*_10_(neutrophils)+*$\sum_{l=1}^{L} \gamma_{l}C_{l}$

where the term $\sum_{l=1}^{L} \gamma_{l}C_{l}$ includes factors estimated to control for batch effects.

The predictor of interest was an indicator of group membership (Control, ADEH- or ADEH+) for each pair-wise comparison of groups. Analyses were performed using multiple testing correction at both a genome-wide level and a candidate gene level, using a set of 129 CpG’s that mapped to a set of genes previously shown to be of interest in EH, specifically *FLG, LCE1B, RPTN, IL4, IL13* and its receptors, *IFNs* and *TSLP*.

***Detection of differentially methylated positions (DMPs) by group without eosinophil adjustment***

We fit a linear model on the M-value scale. Covariates included age, sex and batch, as well as estimates of six cell-type fractions (with eosinophils and neutrophils represented by the granulocyte estimate) and batch effect factors (estimated by RUVm). More specifically our model was of the form:

*DNAm(M-value) ~* β*_0_+*β*_1_(Predictor of interest)+*β*_2_(Sex)+*β*_3_(Age)+*β*_4_(CD8T)+*β*_5_(CD4T)+*β*_6_(NK)+*β*_7_(B cell)+*β*_8_(monocytes)+*β*_9_(granulocytes) +*$\sum_{l=1}^{L} \gamma_{l}C_{l}$

where the term $\sum_{l=1}^{L} \gamma_{l}C_{l}$ includes factors estimated to control for batch effects. The predictor of interest was an indicator of group membership (Control, ADEH- or ADEH+) for each pair-wise comparison of groups. Analyses were performed using multiple testing correction at a genome-wide level.

***Detection of differentially methylated positions by severity***

We fit a linear model on the M-value scale. Covariates included age, sex and batch, as well as estimates of six cell-type fractions (with eosinophils and neutrophils represented by the granulocyte estimate) and batch effect factors (estimated by RUVm). More specifically our model was of the form:

*DNAm(M-value) ~* β*_0_+*β*_1_(Predictor of interest)+*β*_2_(Sex)+*β*_3_(Age)+*β*_4_(CD8T)+*β*_5_(CD4T)+*β*_6_(NK)+*β*_7_(B cell)+*β*_8_(monocytes)+*β*_9_(granulocytes) +*$\sum_{l=1}^{L} \gamma_{l}C_{l}$

where the term $\sum_{l=1}^{L} \gamma_{l}C_{l}$ includes factors estimated to control for batch effects. Two analyses were performed using severity phenotypes: (1) relationship of tIgE levels to methylation in 26 CpGs in the *IL4*, *IL13* and *IL4R* genes to test a specific hypothesis of the role of these genes on tIgE; and (2) relationship of four AD severity measures, including eosinophil counts, total serum IgE (tIgE) levels, EASI score and Rajka-Langeland score to methylation at the subset of CpGs significant from the group-wise genome-wide comparison with six cell types (without adjustment for eosinophil fractions).

***P-value distribution adjustment and multiple testing correction***

A conventional GWAS analysis uses QQ plots as a standard to examine the distribution of P values with a target lambda of 1 being an indicator of proper Type-I error control. It has been argued that this approach is not appropriate in the context of a methylation or expression analysis (11), and a recent paper (11) presents a method for controlling bias and inflation that is specific to EWAS. This method is implemented in the Bioconductor package called *bacon*.

The results obtained from the missMethyl package are then adjusted using *bacon*, to remove inflation and bias often observed in epigenome- and transcriptome-wide association studies. To this end, *bacon* constructs an empirical null distribution using a Gibbs Sampling algorithm by fitting a three-component normal mixture on z-scores (11). P-values output from *bacon* are then FDR-adjusted to account for the number of tests in the specific analysis being performed using the function *p.adjust*.

***Gene-ontology (GO) enrichment analysis***

Gene-ontology enrichment analysis was performed with the *gometh* function in missMethyl, which is designed specifically to address potential biases in measuring gene-set enrichment with the 450K methylation array (12), where probes are not evenly spaced with respect to genes of different types. This method is an adaptation of the goseq method of Young et al. (13), which uses a hypergeometric test to assess enrichment of genes in a particular ontology category among genes closest to significant DMR results. In this adaptation, each gene has a prior probability associated with it based on the number of probes on the array in proximity to the gene.

**Supplementary Figure S1.** Quality control (QC) plot of methylated to unmethylated median intensities for discovery and replication data sets.

Colors indicate processing batch membership.

Discovery data set Replication data set

**Supplementary Figure S2.** Plot of chrX vs chrY median intensities to identify gender mismatches for discovery and replication data sets. Colors indicate sample processing batch. Shapes indicate sex from sample data file.

Discovery data set Replication data set

**Supplementary Figure S3.** QQ plots of p-values from DMP analysis across three phenotype groups (adjusted for age, sex, batch and cell types (7 cell types top 2 rows, 6 for bottom two rows)) prior to and post bacon adjustment.


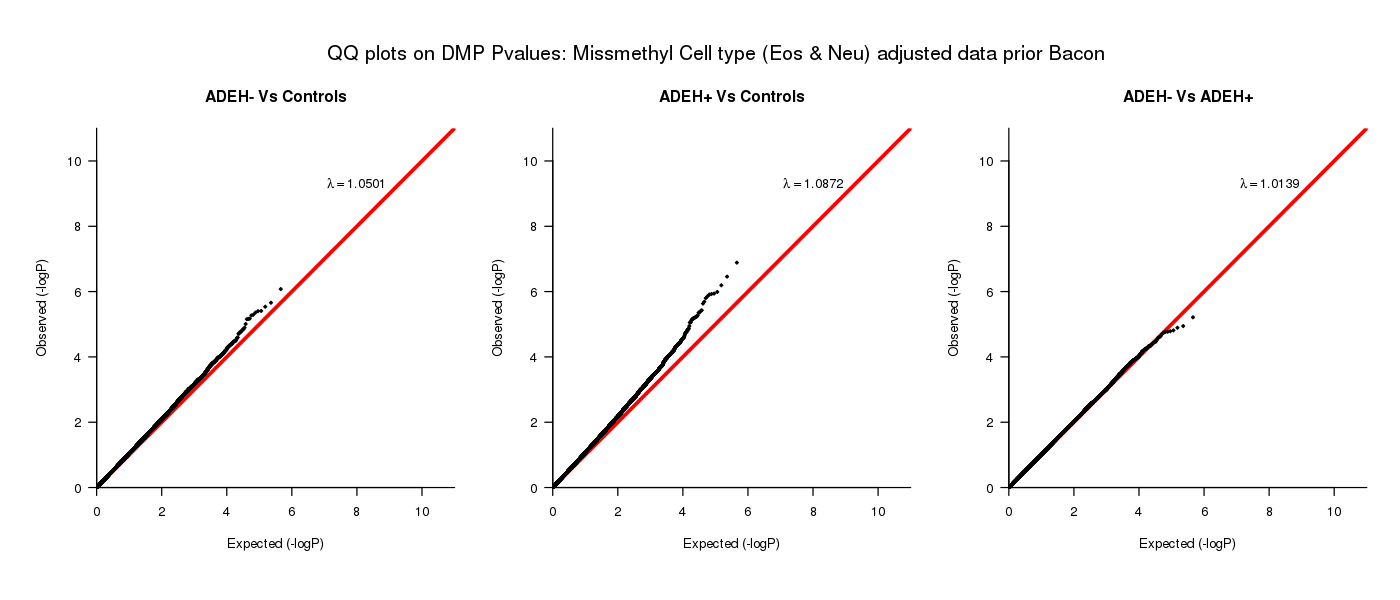


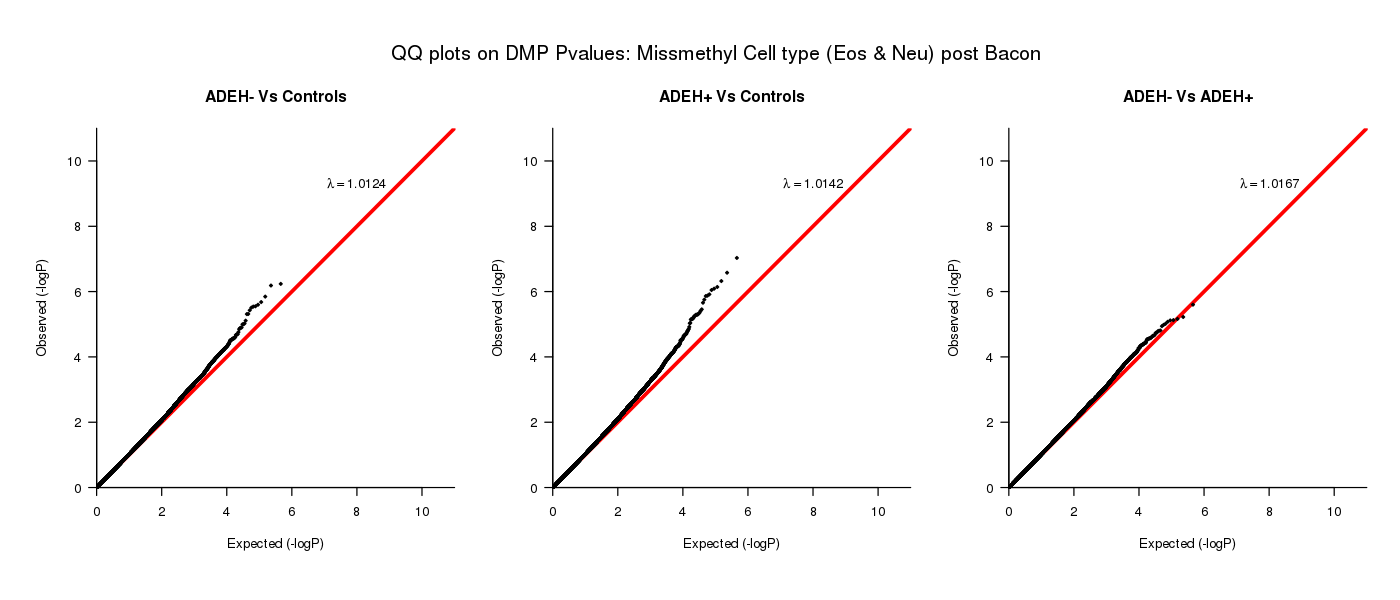


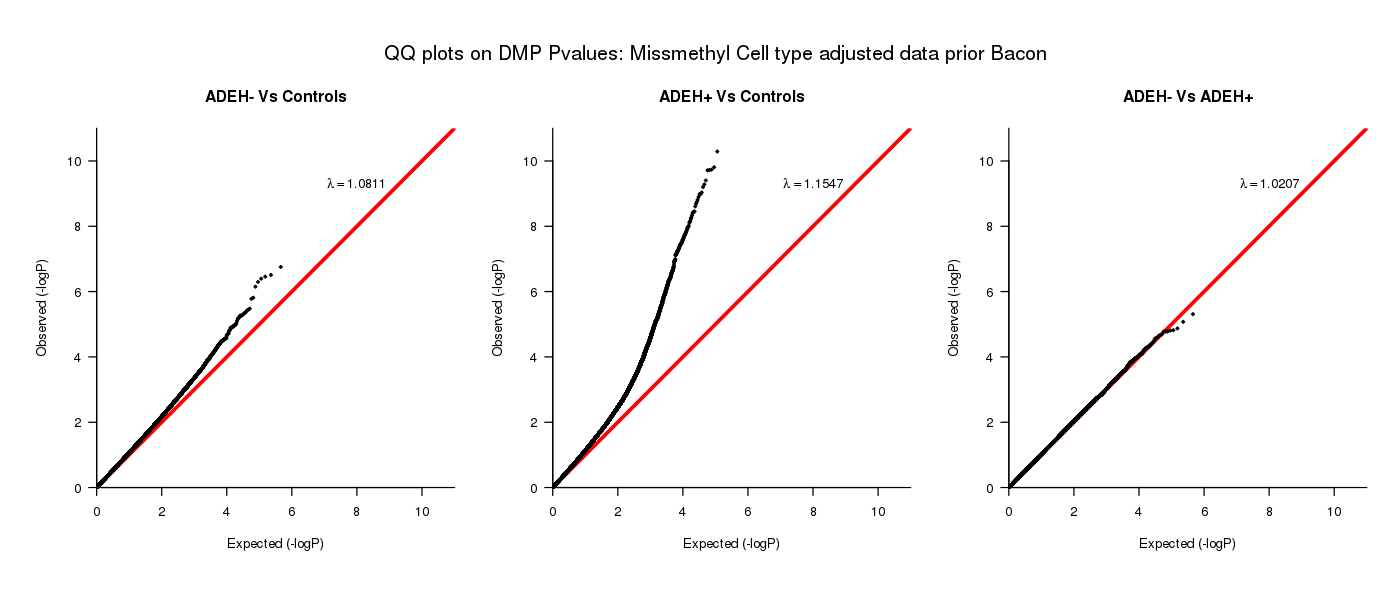


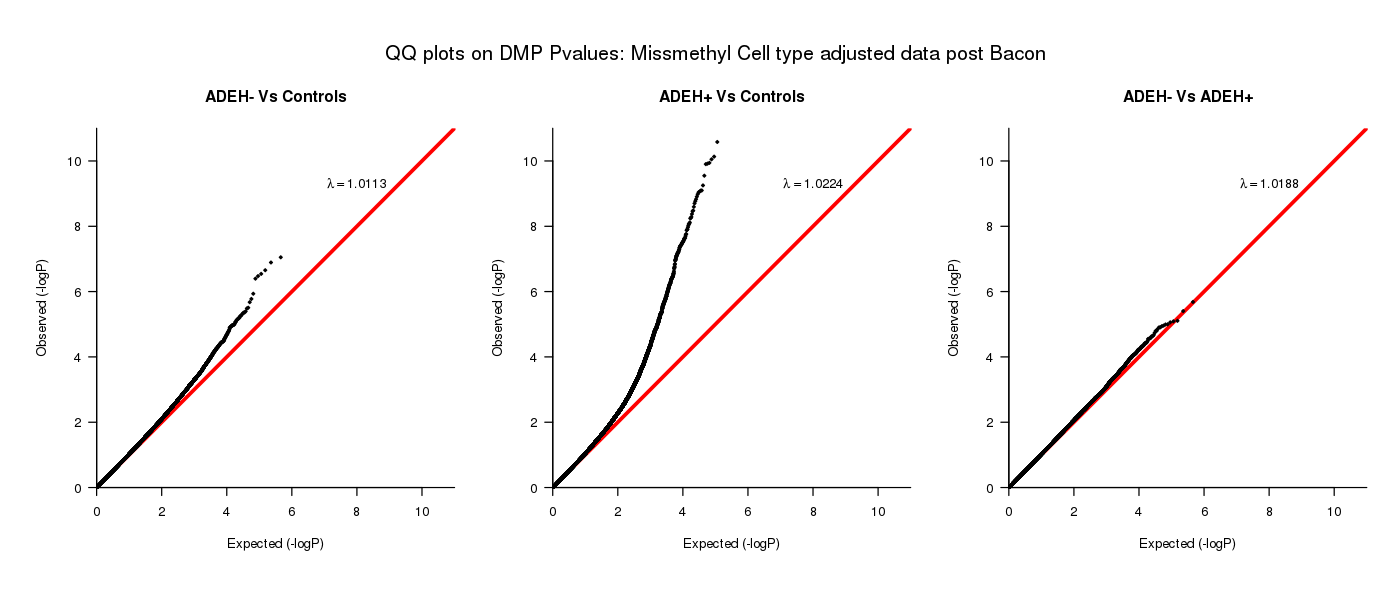


**Supplementary Figure S4:** QQ plots for p-values from DMP analysis for severity phenotypes (adjusted for age, sex, batch and cell types (7 cell types top row, 6 for bottom row, rows paired by phenotype)) prior and post bacon adjustment.


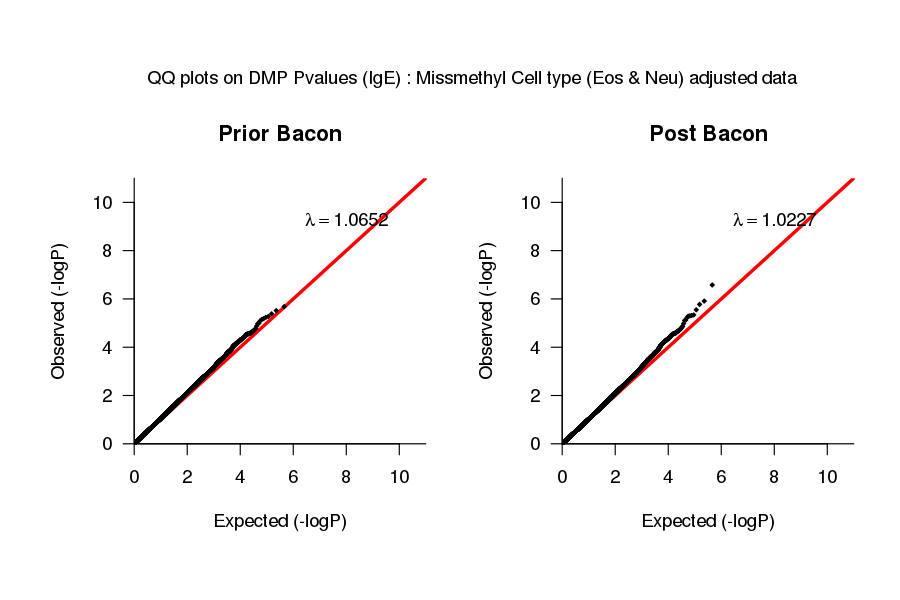


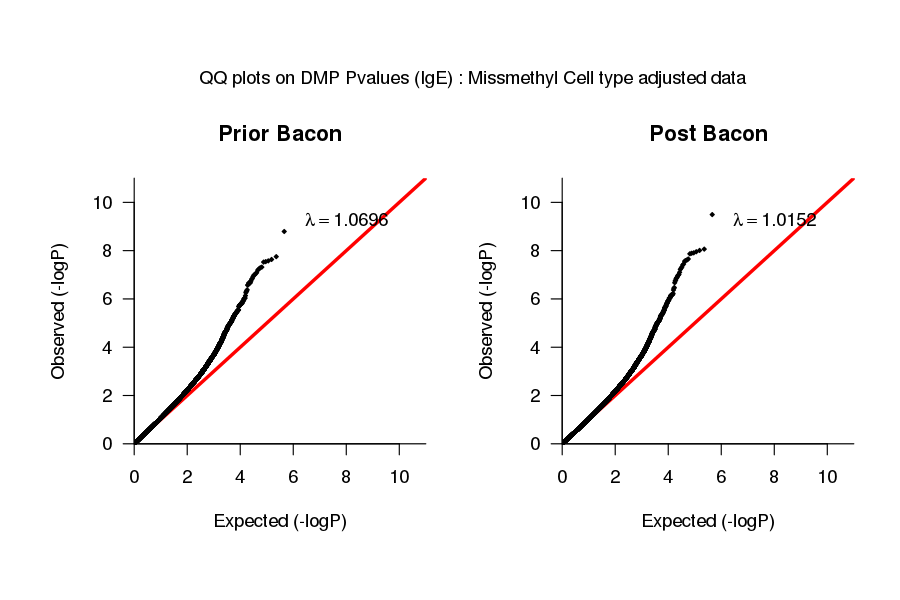


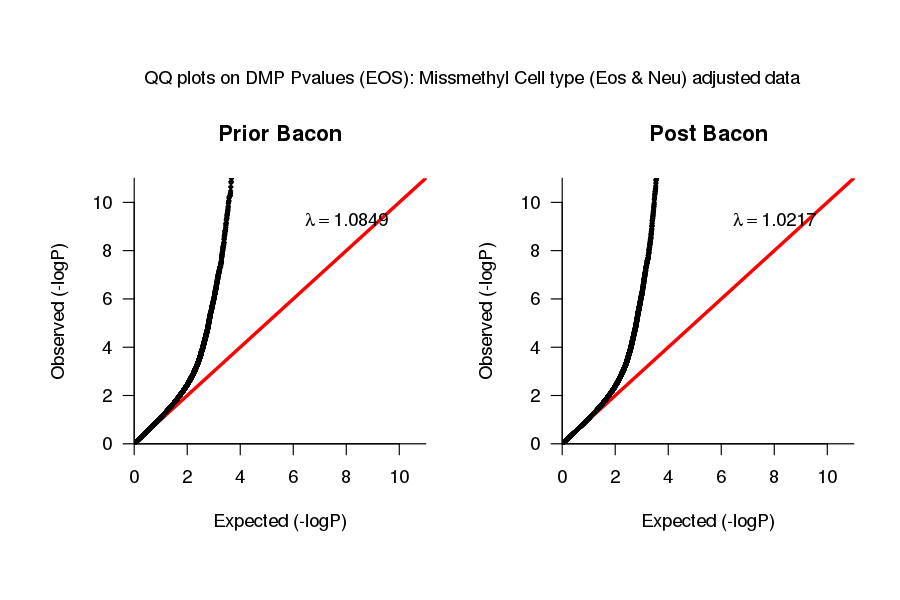


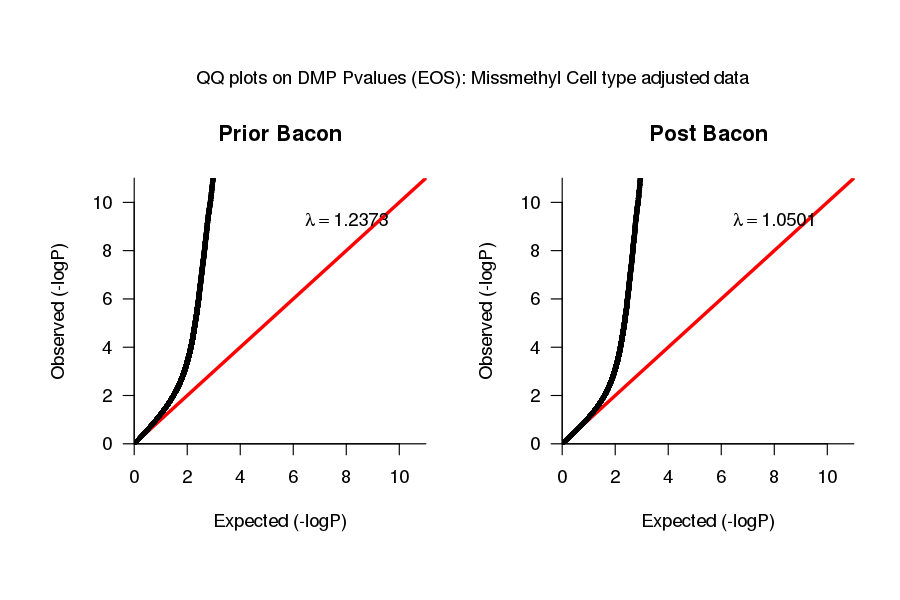


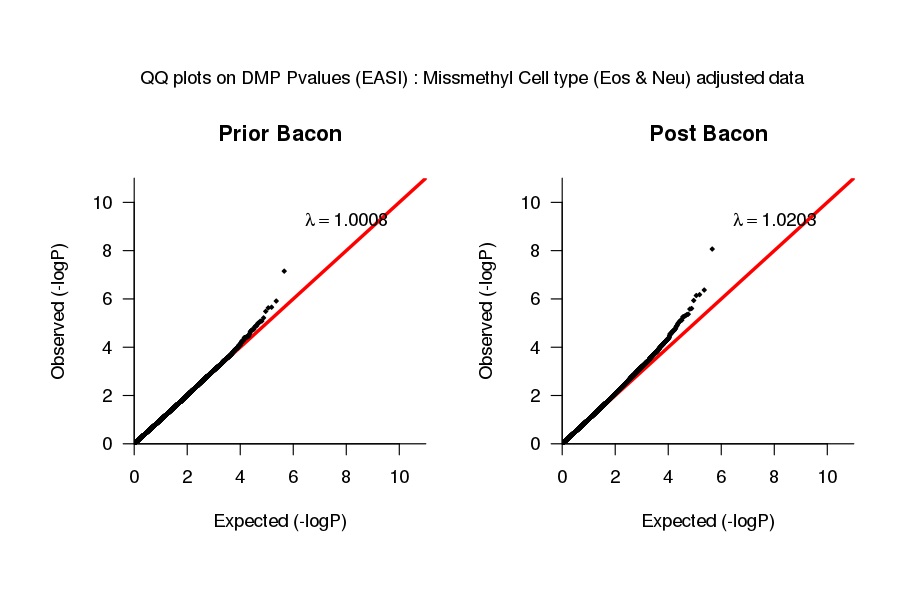


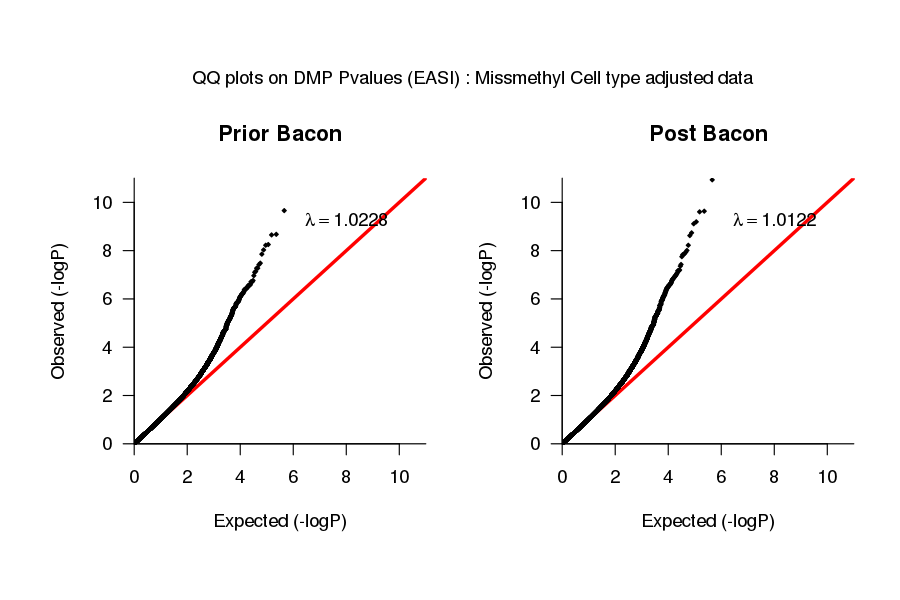


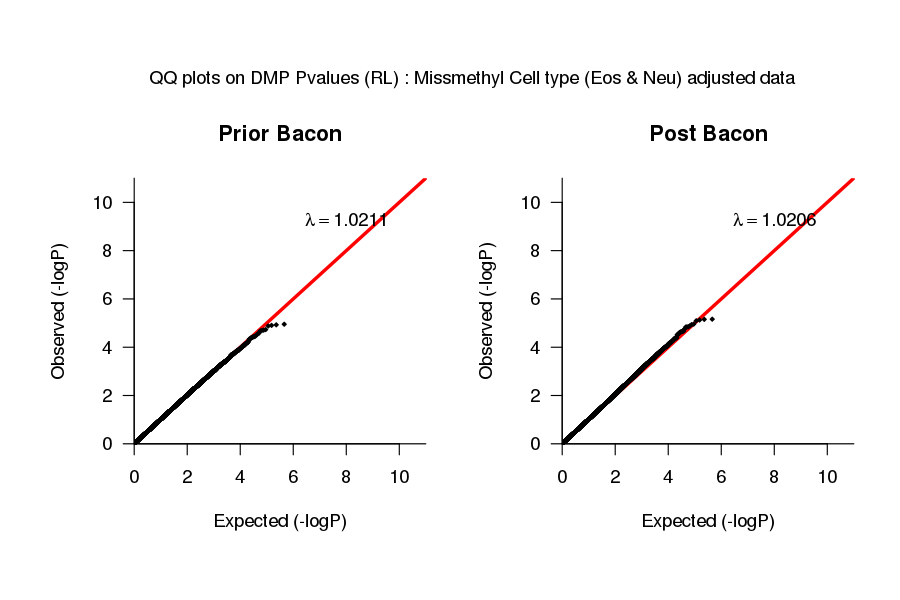


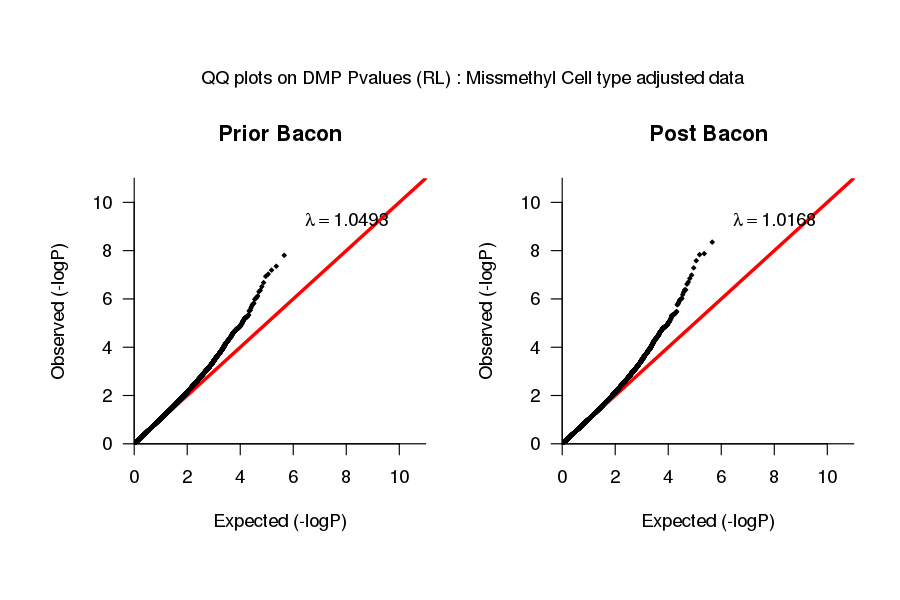


**Supplementary Figure S5**: Boxplots for the top 27 CpG’s significant in ADEH+ Vs controls analysis from model with six cell types adjusted for.

**Supplementary Figure S6.** Scatter plots of top 27 CpGs significant in eosinophil-methylation analysis showing eosinophil levels against methylation values.

**Supplementary Table S1.** Clinical Characteristics table for all samples in discovery and replication data sets

| **Discovery data set (N=297)** | | | |  | **Replication data set (N=167)** | | |
| --- | --- | --- | --- | --- | --- | --- | --- |
| **Trait** | **ADEH+** | **ADEH-** | **Non-atopic controls** |  | **ADEH+** | **ADEH-** | **Non-atopic controls** |
| **N** | **99** | **100** | **98** |  | **56** | **55** | **56** |
| Males (N; %) | 45 (45.5%) | 36 (36%) | 40 (40.8%) |  | 32 (57.1%) | 30 (54.5%) | 19 (33.9%) |
| Age (in years) mean; SD | 28.9; 20.7 | 34.4;14 | 33.2;11.2 |  | 22.1; 20.9 | 24.5; 18.0 | 39.7; 15.7 |
| Total IgE (kU/L) | 1162.9 | 267.5 | 23.7 |  | 1264.1 | 217.4 | 10.8 |
| (95% CI) **^Φ^** | (777.4-1739.5) | (168.5-424.5) | (18.2-30.9) |  | (767.7-2081.5) | (113.7-415.8) | (8.4-13.8) |
|  |  |  |  |  |  |  |  |
| Eosinophils (cells/mm^3^) | 368.7 | 266.1 | 128.7 |  | 370.9 | 269.1 | 89.3 |
| (95% CI) **^Φ^** | (310.5-437.8) | (226.6-312.4) | (113.8-145.7) |  | (298.0-461.7) | (208.8-346.7) | (73.0-109.4) |
| EASI; mean | 12.3 | 10 | (-) |  | 15.2 | 14.5 | (-) |
| (range) | (0-52.4) | (0-66.6) |  |  | (0.9-55.0) | (0.3-47.2) |  |
| Rajka-Langeland mean(SD) | 6.8(1.5) | 6(1.6) | (-) |  | 7.0 (1.4) | 6.9 (1.5) | (-) |
|  |  |  |  |  |  |  |  |
| **^Φ^**Geometric mean |  |  |  |  |  |  |  |

**Supplementary Table S10**. References to support the selection of genes for our gene based analysis

| **Gene** | **References** |
| --- | --- |
| *FLG* | (14-19) |
| *LCE1B* | (19, 20) |
| *RPTN* | (20, 21) |
| *IL4* | (16, 19, 22-25) |
| *IL13* | (16, 19, 22-25) |
| *IFN* | (26-28) |
| *TSLP* | (16, 19, 22, 29, 30) |

**References:**

1. Weisenberger DJ VDBD, Pan F, Berman BP, and Laird PW. Comprehensive DNA Methylation Analysis on the Illumina Infinium Assay Platform. Illumina application note. 2008.

2. Gunderson KL, Steemers FJ, Lee G, Mendoza LG, Chee MS. A genome-wide scalable SNP genotyping assay using microarray technology. Nature Genetics. 2005;37(5):549-54.

3. Du P, Zhang X, Huang C-C, Jafari N, Kibbe WA, Hou L, et al. Comparison of Beta-value and M-value methods for quantifying methylation levels by microarray analysis. BMC Bioinformatics. 2010;11(1):587.

4. Hanifin JM, Thurston M, Omoto M, Cherill R, Tofte SJ, Graeber M, et al. The eczema area and severity index (EASI): assessment of reliability in atopic dermatitis. Experimental Dermatology. 2001;10(1):11-8.

5. Rajka G, Langeland T. Grading of the severity of atopic dermatitis. Acta Derm Venereol Suppl (Stockh). 1989;144:13-4.

6. Jaffe AE, Irizarry RA. Accounting for cellular heterogeneity is critical in epigenome-wide association studies. Genome Biology. 2014;15(2):R31.

7. Aryee MJ, Jaffe AE, Corrada-Bravo H, Ladd-Acosta C, Feinberg AP, Hansen KD, et al. Minfi: a flexible and comprehensive Bioconductor package for the analysis of Infinium DNA methylation microarrays. Bioinformatics. 2014;30(10):1363-9.

8. Houseman E, Accomando WP, Koestler DC, Christensen BC, Marsit CJ, Nelson HH, et al. DNA methylation arrays as surrogate measures of cell mixture distribution. BMC Bioinformatics. 2012;13(1):86.

9. Gagnon-Bartsch JA JL, Speed TP. Removing Unwanted Variation from High Dimensional Data with Negative Controls. Tech Reports from Dep Stat Univ California, Berkeley. 2013:1–112.

10. Maksimovic J, Gagnon-Bartsch JA, Speed TP, Oshlack A. Removing unwanted variation in a differential methylation analysis of Illumina HumanMethylation450 array data. Nucleic Acids Research. 2015;43(16):e106-e.

11. van Iterson M, van Zwet EW, Heijmans BT. Controlling bias and inflation in epigenome- and transcriptome-wide association studies using the empirical null distribution. Genome Biology. 2017;18(1).

12. Geeleher P, Hartnett L, Egan LJ, Golden A, Raja Ali RA, Seoighe C. Gene-set analysis is severely biased when applied to genome-wide methylation data. Bioinformatics. 2013;29(15):1851-7.

13. Young MD, Wakefield MJ, Smyth GK, Oshlack A. Gene ontology analysis for RNA-seq: accounting for selection bias. Genome Biology. 2010;11(2):R14.

14. Beck LA, Boguniewicz M, Hata T, Schneider LC, Hanifin J, Gallo R, et al. Phenotype of atopic dermatitis subjects with a history of eczema herpeticum. Journal of Allergy and Clinical Immunology. 2009;124(2):260-9.e7.

15. Gao P-S, Leung DYM, Rafaels NM, Boguniewicz M, Hand T, Gao L, et al. Genetic Variants in Interferon Regulatory Factor 2 (IRF2) Are Associated with Atopic Dermatitis and Eczema Herpeticum. Journal of Investigative Dermatology. 2012;132(3):650-7.

16. Kim KW, Myers RA, Lee JH, Igartua C, Lee KE, Kim YH, et al. Genome-wide association study of recalcitrant atopic dermatitis in Korean children. Journal of Allergy and Clinical Immunology. 2015;136(3):678-84.e4.

17. Ziyab AH, Karmaus W, Holloway JW, Zhang H, Ewart S, Arshad SH. DNA methylation of the filaggrin gene adds to the risk of eczema associated with loss-of-function variants. Journal of the European Academy of Dermatology and Venereology. 2012;27(3):e420-e3.

18. Boguniewicz M, Leung DYM. Atopic dermatitis: a disease of altered skin barrier and immune dysregulation. Immunological Reviews. 2011;242(1):233-46.

19. Bin L, Leung DYM. Genetic and epigenetic studies of atopic dermatitis. Allergy, Asthma & Clinical Immunology. 2016;12(1).

20. Baurecht H, Hotze M, Brand S, Büning C, Cormican P, Corvin A, et al. Genome-wide Comparative Analysis of Atopic Dermatitis and Psoriasis Gives Insight into Opposing Genetic Mechanisms. The American Journal of Human Genetics. 2015;96(1):104-20.

21. Trzeciak M, Sakowicz-Burkiewicz M, Wesserling M, Gleń J, Dobaczewska D, Bandurski T, et al. Altered Expression of Genes Encoding Cornulin and Repetin in Atopic Dermatitis. International Archives of Allergy and Immunology. 2017;172(1):11-9.

22. Boguniewicz M, Leung DYM. Recent insights into atopic dermatitis and implications for management of infectious complications. Journal of Allergy and Clinical Immunology. 2010;125(1):4-13.

23. Potaczek DP, Kabesch M. Current concepts of IgE regulation and impact of genetic determinants. Clin Exp Allergy. 2012;42(6):852-71.

24. Beck LA, Thaçi D, Hamilton JD, Graham NM, Bieber T, Rocklin R, et al. Dupilumab Treatment in Adults with Moderate-to-Severe Atopic Dermatitis. New England Journal of Medicine. 2014;371(2):130-9.

25. Oettgen HC. Fifty years later: Emerging functions of IgE antibodies in host defense, immune regulation, and allergic diseases. J Allergy Clin Immunol. 2016;137(6):1631-45.

26. Leung DYM, Gao P-S, Grigoryev DN, Rafaels NM, Streib JE, Howell MD, et al. Human atopic dermatitis complicated by eczema herpeticum is associated with abnormalities in IFN-γ response. Journal of Allergy and Clinical Immunology. 2011;127(4):965-73.e5.

27. Bin L, Edwards MG, Heiser R, Streib JE, Richers B, Hall CF, et al. Identification of novel gene signatures in patients with atopic dermatitis complicated by eczema herpeticum. Journal of Allergy and Clinical Immunology. 2014;134(4):848-55.

28. Ovsyannikova IG, Haralambieva IH, Kennedy RB, O'Byrne MM, Pankratz VS, Poland GA. Genetic Variation in IL18R1 and IL18 Genes and Inteferon γ ELISPOT Response to Smallpox Vaccination: An Unexpected Relationship. The Journal of Infectious Diseases. 2013;208(9):1422-30.

29. Cianferoni A, Spergel J. The importance of TSLP in allergic disease and its role as a potential therapeutic target. Expert Review of Clinical Immunology. 2014;10(11):1463-74.

30. Gao P-S, Rafaels NM, Mu D, Hand T, Murray T, Boguniewicz M, et al. Genetic variants in thymic stromal lymphopoietin are associated with atopic dermatitis and eczema herpeticum. Journal of Allergy and Clinical Immunology. 2010;125(6):1403-7.e4.
